# Specific test panels for patients with heart failure: implementation and use in the Spanish National Health System

**DOI:** 10.1515/almed-2022-0006

**Published:** 2022-03-07

**Authors:** Luis Almenar Bonet, Mᵃ Teresa Blasco Peiró, Begoña Laiz Marro, Miguel Camafort Babkowski, Antonio Buño Soto, Maria Generosa Crespo-Leiro

**Affiliations:** Unit of Heart Failure and Transplant, Service of Cardiology, University and Polytechnic La Fe Hospital of Valencia, Valencia, Spain; University of Valencia, Valencia, Spain; Spanish Network-Center for Cardiovascular Biomedical Research (CIBERCV), Madrid, Spain; Unit of Heart Failure and Transplant, Service of Cardiology, Miguel Servet University Hospital, Zaragoza, Spain; University of Zaragoza, Zaragoza, Spain; Laboratory Analysis Service, University and Polytechnic La Fe Hospital of Valencia, Valencia, Spain; Service of Internal Medicine, ICMiD, Hospital Clínic, Barcelona, Spain; University of Barcelona, Barcelona, Spain; August Pi i Sunyer Biomedical Research Institute (IDIBAPS), Barcelona, Spain; Laboratory Analysis Service, La Paz University Hospital, Madrid, Spain; Unit of Heart Failure and Heart Transplant, Service of Cardiology, A Coruña Hospital Complex, CHUAC, A Coruña (UDC), Spain; Biomedical Research Institute of A Coruña (INIBIC), A Coruña, Spain; University of A Coruña, A Coruña, Spain; Centro de Investigación Biomédica en Red de Enfermedades Cardiovasculares (CIBERCV), Madrid, Spain

**Keywords:** diagnosis, healthcare management, heart failure, iron profile, monitoring, test panels

## Abstract

**Objectives:**

The use of specific test panels (STP) for heart failure (HF) could help improve the management of this condition. The purpose of this study is to gain an insight into the level of implementation of STPs in the management of HF in Spain and gather the opinions of experts, with a special focus on parameters related to iron metabolism.

**Methods:**

The opinions of experts in HF were gathered in three stages STAGE 1 as follows: level of implementation of STPs (n=40). STAGE 2: advantages and disadvantages of STPs (n=12). STAGE 3: level of agreement with the composition of three specific STPs for HF: initial evaluation panel, monitoring panel, and *de novo* panel (n=16).

**Results:**

In total, 62.5% of hospitals used STPs for the clinical management of HF, with no association found between the use of STPs and the level of health care (p=0.132) and location of the center (p=0.486) or the availability of a Heart Failure Unit in the center (p=0.737). According to experts, the use of STPs in clinical practice has more advantages than disadvantages (8 vs. 3), with a notable positive impact on diagnostics. Experts gave three motivations and found three limitations to the implementation of STPs. The composition of the three specific STPs for HF was viewed positively by experts.

**Conclusions:**

Although the experts interviewed advocate the use of diagnostic and monitoring STPs for HF, efforts are still necessary to achieve the standardization and homogenization of test panels for HF in Spanish hospitals.

## Introduction

Specific test panels (STPs) are useful to prevent the overutilization and underutilization of laboratory tests and counter the increased demand and cost of healthcare resources [1, 2]. When adjusted to different levels of health care and settings, STPs improve disease control and resource use, reduce costs, homogenize healthcare, and facilitate research.

Heart failure (HF) is a clinical syndrome causing shortness of breath, ankle swelling and fatigue which signs include pulmonary crackles, elevated jugular venous pressure and peripheral edema [3]. This pathological condition is the consequence of an anomaly in cardiac structure or function and is considered a major public health problem [3]. The prevalence of HF in adults in developed countries is 2%, increasing dramatically with age, being <1% before 50 years, doubling with each decade, and reaching 8% above 75 years [3]. HF is the leading cause of hospitalization in patients older than 65 and accounts for more than 2% of healthcare expense in Spain. It is also a frequent cause of morbidity and mortality [3], [4], [5], [6]. The etiology of HF is multifactorial, with several risk factors being involved [7]. Since iron deficiency is common in patients with HF [8], [9], [10], routine iron profile testing (serum ferritin and transferrin saturation) is recommended for all patients with suspected or confirmed HF [3]. Including iron tests in STPs for HF would ensure compliance with this recommendation. STPs may facilitate the diagnosis and monitoring of HF and its comorbidities. This would improve healthcare quality and clinical outcomes and reduce the direct costs of unnecessary testing and the indirect costs of failing to order appropriate tests. Our primary goal was to evaluate the level of implementation of STPs for HF, a prevalent condition in Spain. Secondary goals included exploring the advantages and disadvantages of STP; assessing whether STPs include the parameters indicated for HF – including iron profile – in the guidelines and interviewing experts in HF about the suitability of three STPs for HF.

## Materials and methods

### Study design and data collection

The methodological design and field work were performed by the medical agency (MA) Anima Strategic Consulting. Non-probability purposeful sampling was employed to define the study population and perform participant recruitment, taking into account sample dispersion by level of health care, geographical location, and presence of a Heart Failure Unit (HFU) in the center. Emergency care was excluded.

Informed consent was obtained before interviews. The first stage (STAGE 1) included computer-assisted telephone interviewing (CATI) to gather the opinions of cardiologists from different regions in Spain (November–December 2019). STAGE 1 involved a prospective study of the level of automation of laboratory tests and the creation and use of STPs for HF. CATI questionnaires were jointly designed with the MA with yes/no answers. Participants in STAGE 1 were cardiologists with more than five years of experience after their residency attending at least 15 HF patients monthly. Geographical locations, main characteristics, and level of health care of each center according to World Health Organization classification [11] are shown in Supplementary Figure 1. The questionnaire is available in Supplementary Figure 2.

In STAGE 2, experts in STPs were interviewed (May–June 2020). The panel was composed of seven expert cardiologists in HF whose hospitals had integrated a STP for HF, and five clinical laboratory directors (three hospital and two external laboratories) experienced in creating STPs (Supplementary Figure 3). We collected information about STP design process and identified best practices in their implementation in centers of reference. Owing to COVID-19, interviews, designed and conducted by the MA, were performed by video call (see questionnaire in Supplementary Figure 4).

In STAGE 1, the interviewers obtained the results of the CATI questionnaire in real time and, for quality control, reviewed the recordings to verify information. Interviews were then evaluated by a different investigator. Subsequently, another investigator reviewed the analysis of 10 random interviews. Field work results were obtained by joint cross-interview analysis.

STAGE 2 interviews were transcribed verbatim and analyzed as in STAGE 1, except that, for quality purposes, interviews were conducted and transcribed by different consultants. Qualitative analysis was performed by two investigators, who classified and shared answers. Following classification, the same investigators counted the repetitions of each answer.

In STAGE 3, the authors designed three STPs for the clinical management of HF [initial evaluation panel, monitoring panel, and *de novo* panel (see Supplementary Table 1)]. These STPs were constructed considering the latest scientific evidence and the most commonly assayed laboratory parameters at these three key time points. Sixteen experts in HF discussed the composition and efficacy of these parameters and advocated or opposed to the inclusion of each parameter.

### Statistical analysis

Qualitative variables are expressed in absolute values. Differences between groups were analyzed using Chi-square test or Fisher’s exact test. Statistical analysis was performed using GraphPad Prism 9.0 (GraphPad Software, Inc., San Diego, CA).

## Results

### Level of implementation of STPs for HF

All hospitals had a laboratory information system and used different laboratory analysis request protocols.

Of the 40 hospitals analyzed, 15 (37.5%) had not established a STP for HF, therefore parameters were selected individually (Table 1), whereas 25 (62.5%) employed a specific STP for HF. With just a click, HF-specific parameters were selected.

**Table 1:** Types of tests performed in patients with HF by level of health care.

			STP	Individualized	p-Value
Level of health care	n (%)	Primary	3 (7.5)	0	0.132
		Secondary	8 (20)	9 (22.5)	
		Tertiary	14 (35)	6 (15)	
Location	n (%)	Rural	8 (20)	3 (7.5)	0.486
		Urban	17 (42.5)	12 (30)	
HFU	n (%)	With	8 (20)	6 (15)	0.737
		Without	17 (42.5)	9 (22.5)	
Total	n (%)		25 (62.5)	15 (37.5)	

HF, heart failure; STP, specific test panel; Individualized, individualized selection system.

STAGE 1 did not provide evidence of a correlation between level of automation of laboratory test orders and level of health care (p=0.132; tertiary, secondary or primary level), location (p=0.486; urban or rural) or the presence/absence of a HFU (p=0.737; Table 1).

Supplementary Table 2 contains data on the presence of iron profile (IP) tests in STPs or in pre-established parameter sets (in hospitals without STP). IP testing was associated with tertiary level (p=0.048). Neither location nor the availability of a HFU were associated with IP testing in HF patients.

### Advantages and disadvantages of STPs

**Figure 1:**
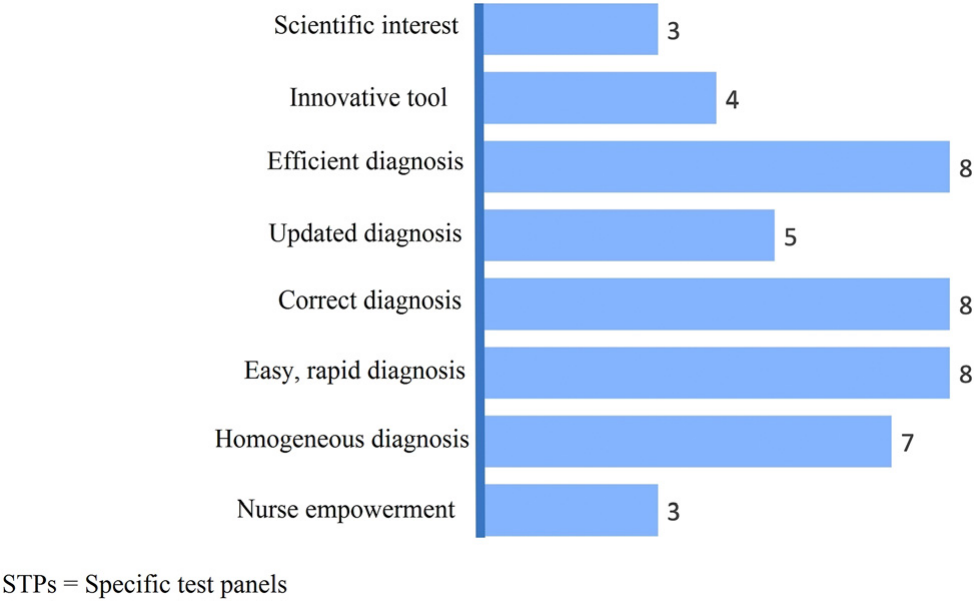
Advantages of STPs.

In STAGE 2, cardiologists and laboratory directors described the advantages of STPs (Figure 1), including their scientific relevance, innovative nature, capacity to empower nurses and facilitate effective, correct updated, homogeneous, easy and rapid diagnosis. The most frequently cited advantages were that STPs enable effective, correct easy and rapid diagnosis (eight votes) and homogeneous diagnosis (seven votes).

**Figure 2:**
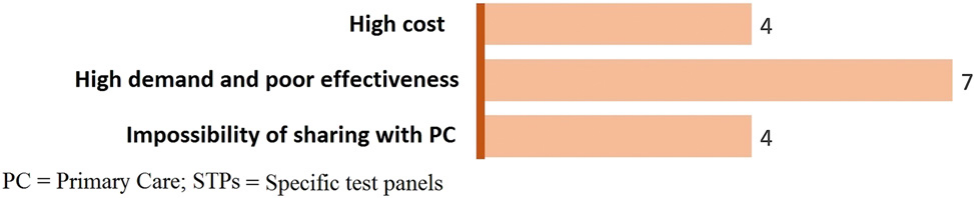
Disadvantages of STPs.

Disadvantages include a poorer cost-effectiveness if STPs are not correctly designed, the impossibility of sharing them with primary care, and its high demand and low efficacy, being the latter the most frequently cited disadvantage (seven votes; Figure 2).

### Incentives and barriers to the establishment of STPs

**Figure 3:**
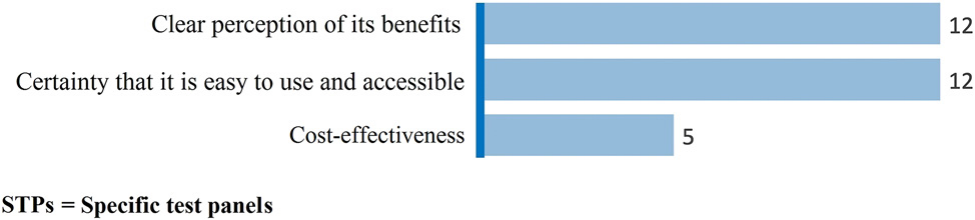
Reasons for the implementation of STPs.

Motivations for the implementation of STPs included their cost-effectiveness, a clear perception of its benefits, and the simplicity and accessibility of the system (Figure 3), being the last two answers the most frequently cited advantages.

**Figure 4:**
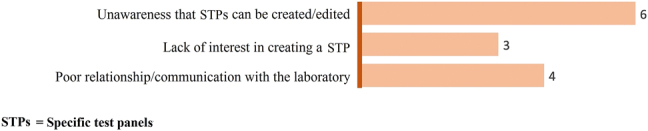
Barriers to the implementation of STPs.

By increasing order of frequency, drawbacks of STPs (Figure 4) include lack of interest in creating a STP, poor relationship and/or communication with the laboratory, and unawareness of the possibility of creating and editing STPs.

### A STPs model for the clinical management of HF: the opinions of experts

Table 2 contains the three STPs models developed for HF. Three of the experts consulted (18.75%) did not consider STPs for HF necessary. Two experts had already created other test panels and did not deem it necessary to create new test panels. The other expert opposing to STPs argued that his Cardiology Unit used a general cardiology laboratory profile, which he considered suitable for the clinical management of HF. Most of the 13 experts (81.25%) advocating the use of the three STPs for HF considered their composition suitable. Regarding Panel 1 (initial evaluation profile), experts unanimously agreed with all the parameters included, except for CA-125 (five opposing votes) and NT-proBNP (two opposing votes). All experts coincided that iron profile tests should be included in Panel 1. There was also unanimity on most of the parameters of Panel 2 (monitoring profile). In this case, two experts opposed to the inclusion of uric acid and NT-proBNP in this profile. Finally, the majority of experts agreed with Panel 3 (*de novo* profile) composition, although unanimity was only reached on urine.

**Table 2:** STP model for the clinical management of patients with HF and opinions of experts.

PANEL 1: Initial evaluation test panel		
	In favor	Against
Glycosylated hemoglobin A1c	13	0
Urea	13	0
Glucose	13	0
Creatinine	13	0
Uric acid	13	0
Total cholesterol	13	0
HDL cholesterol (direct)	13	0
LDL cholesterol (calculated)	13	0
Triglycerides	13	0
AST/GOT	13	0
ALT/GPT	13	0
GGT	13	0
Alkaline phosphatase	13	0
Total bilirubin	13	0
Total proteins	13	0
Albumin	13	0
Ions (Na, K, Cl)	13	0
Ferritin	13	0
Transferrin saturation index (TSI)	13	0
CA125	8	5
NT-proBNP	11	2
Prothrombin time	13	0
INR	13	0
Hemogram	13	0

PANEL 2: Monitoring test panel		
	In favor	Against
Urea	13	0
Glucose	13	0
Creatinine	13	0
Uric acid	11	2
AST/GOT	13	0
ALT/GPT	13	0
GGT	13	0
Alkaline phosphatase	13	0
Total bilirubin	13	0
Total proteins	13	0
Albumin	13	0
Ions (Na, K, Cl)	13	0
NT-proBNP	11	2
Prothrombin time	13	0
INR	13	0
Hemogram	13	0

PANEL 3: *De novo* test panel		
	In favor	Against
Thyroid hormones (TSH)^a^	11	2
High-sensitivity troponin (T or I)	12	1
CRP (C reactive protein)	12	1
Urine metanephrines^b^	11	2
Urine (sediment abnormalities, proteinuria)	13	0

HF, heart failure; STPs, specific test panels. ^a^Free T4 and free T3 in the presence of TSH alteration. ^b^Consider indication.

## Discussion

Healthcare management is undergoing the digitization, automation and standardization of processes [12]. Developed countries have experienced a dramatic rise in health burden and costs, and this tendency is estimated to maintain in the near future [1]. The effective use of standard automated test panels may help reduce costs and improve the clinical management of patients.

This study demonstrates differences in the management and use of laboratory tests and iron profile testing in HF. STPs were available in 62.5% of hospitals vs. 37.5%. There was no association between the use of STPs and level of health care, location, or HFU availability. There was a statically significant difference between protocols for requesting iron profile tests and level of health care. Real clinical practice is inconsistent with the opinions of experts on the three STPs developed. Experts advocated iron profile testing, since iron deficiency is common in HF. This deficiency is associated with poorer physical performance, deterioration of health-related quality of life and a higher risk of mortality, irrespectively of the associated presence or not of anemia [8], [9], [10, 13]. The 2021 *European Society of Cardiology* (ESC) guidelines for the diagnosis and treatment of HF [3] recommend routinely testing iron profile in all patients with suspected or confirmed HF. When iron deficiency is detected, supplementation is prescribed resulting in clinical benefits for the patient [14], [15], [16], [17]. Iron supplementation in HF is also cost-effective, as demonstrated by Delgado et al. [18]. A meta-analysis conducted by Khan in 2020 [15] and two clinical trials (CONFIRM-HF [17] and AFFIRM-AHF [16]) reveal that the administration of iron carboxymaltose to HF patients reduces the risk of hospitalization, resulting in a reduction of health costs [15], [16], [17]. Therefore, iron profile should be routinely tested in HF patients, which STPs could facilitate, since they help translate best practices into real practice.

Experts found more advantages than disadvantages in the integration of STPs, with improvements in diagnosis being the most frequently cited advantage. STPs were considered an easy-to-use, accessible tool, with the experts interviewed having a clear perception of the benefits of this system. The main drawback was unawareness that test panels can be created and/or edited. Experts advocate the use of STPs and find few barriers to their implementation. However, the use of STPs is far from commonplace, possibly due to the fact that health professionals lack the time to develop and implement new protocols [19, 20]. It is necessary to raise awareness on the need to improve strategic planning, which would have positive effects on healthcare management [19, 20].

This study shows inconsistencies in the use and composition of STPs, which hinders cross-site comparison. All experts agreed with the composition of the three STPs proposed, with some divergences on their composition. There was discrepancy concerning the inclusion of CA-125 in Panel 1. Although there is consistent scientific evidence supporting its inclusion in laboratory tests for HF, it is rarely mentioned in clinical guidelines [21, 22]. There were some minor discrepancies concerning the inclusion of NT-proBNP in Panels 1 and 2. The 2021 ESC guidelines *do* recommend the inclusion of NT-proBNP in the laboratory profile for HF [3]. However, experts did not consider it necessary to test NT-proBNP in all patients. Three experts in STAGE 3 deemed that Panel 3 parameters (thyroid hormones, cardiac troponin measured by high sensitivity method, CRP and plasma metanephrines) should be integrated into Panel 1. Experts would only order a urine test (sediment, proteinuria) in the monitoring laboratory test of hospitalized patients. The three STPs proposed are a starting point in terms of content and number, and may help design consensus recommendations for the use of STPs in HF.

Limitations of this study include sample size, if we consider the number of health professionals involved in the management of HF in Spain. Nevertheless, this sample is representative of health professionals in Spain, as it includes hospitals of different levels of care, locations and with and without HFU. Another limitation is the use of CATI for data collection, which could be affected by the absence of visual signals and data loss and distortion [23, 24]. However, CATI is a widely-accepted data collection tool in clinical practice, public health research and epidemiology, as it enables gathering large data sets and managing sensitive information [23].

In the last decades, efforts have been made to ensure the appropriate use of laboratory tests [25, 26] both in secondary or tertiary care [27, 28] and primary care [29, 30]. Strategies included modifications to electronic laboratory test request systems, targeted training, and feedback about the behavior of laboratory test orders [31], [32], [33], [34], [35], [36]. This study aims to add to these strategies by providing information about the use of STPs for HF in real practice. STPs improve the organizational model for the management of HF patients, following the recommendations of the Spanish Society of Cardiology (SEC), the Spanish Society of Urgencies and Emergencies (SEMES), and the Spanish Society of Internal Medicine (SEMI) [37]. This is the first study to assess STPs in Spain and shows that standardization and homogenization of diagnostic and monitoring tests in patients with HF is still a pending issue. In this work, we developed three STPs for the clinical management of patients with HF and gathered the opinions of experts on three STPs models, with positive results. This could be a starting point for the validation and implementation of STPs for HF. STPs could help establish a standard strategy for monitoring HF in the National Health System, may encourage further research, and could ultimately result in improved clinical outcomes and reduced healthcare costs. Future actions are necessary to promote, encourage and spread the use of STPs for HF.

In conclusion, the implantation of STPs is still very limited in Spain. However, most of the healthcare professionals interviewed consider that STPs are useful for the homogenization and standardization of STPs for HF and advocate their implementation and use. It is necessary to improve communication and coordination between laboratory and cardiology services. Likewise, modern technologies may help improve laboratory test ordering through STPs for the clinical management of HF.

## Supplementary Material

Supplementary Material Details

Supplementary Material Details

Supplementary Material Details

Supplementary Material Details

Supplementary Material Details

Supplementary Material Details
