# Supplementary material for: Specific test panels for patients with heart failure: implementation and use in the Spanish National Health System
Source: Adv Lab Med. 2022 Mar 7;3(1):65–70. doi: 10.1515/almed-2022-0006 (PMC10197348; doi:10.1515/almed-2022-0006)
Supplement: Supplementary file 1 — Supplementary Material Details [file j_almed-2022-0006_suppl_001.docx]

**Supplementary Table 1.**

Panels proposed, test timing, and level of health care.

|  | **Timing** | **Level of health care** |
| --- | --- | --- |
| **Panel 1: Initial evaluation panel** | - First test ordered in ward after hospitalization. - Outpatient follow-up every 3-6 months. | - Hospitalization - Ambulatory |
| **Panel 2: Monitoring panel** | - Follow-up in ward every 2-3 days. - In case of significant change in treatment. | - Hospitalization |
| **Panel 3: *De novo* panel** | - In case of new diagnosis, once the patient is stabilized. | - Hospitalization - Ambulatory |
