# Supplementary material for: Specific test panels for patients with heart failure: implementation and use in the Spanish National Health System
Source: Adv Lab Med. 2022 Mar 7;3(1):65–70. doi: 10.1515/almed-2022-0006 (PMC10197348; doi:10.1515/almed-2022-0006)
Supplement: Supplementary file 2 — Supplementary Material Details [file j_almed-2022-0006_suppl_002.docx]

**Supplementary Table 2.**

Types of tests including iron profile in HF patients by level of health care.

|  |  |  | **STP including IP** | **STP not including IP** | **Individualized, including IP** | **Individualized, not including IP** | ***p*-value** |
| --- | --- | --- | --- | --- | --- | --- | --- |
| **Level of health care** | n(%) | Primary | 1 (2.5) | 2 (5) | 0 (0) | 0 (0) | 0.048* |
|  |  | Secondary | 4 (10) | 4 (10) | 1 (2.5) | 8 (20) |  |
|  |  | Tertiary | 12 (30) | 2 (5) | 3 (7.5) | 3 (7.5) |  |
| **Location** | n(%) | Rural | 4 (30) | 4 (10) | 0 (0) | 3 (7.5) | 0.298 |
|  |  | Urban | 13 (32.5) | 4 (10) | 4 (10) | 8 (20) |  |
| **HFU** | n(%) | With | 3 (7.5) | 5 | 1 (2.5) | 5 | 0.132 |
|  |  | Without | 14 (35) | 3 (7.5) | 3 (7.5) | 6 (15) |  |
| **Total** | n(%) |  | 17 (42.5) | 8 (20) | 4 (10) | 11 (27.5) |  |

STP = Specific test panel; Individualized = individualized selection system; IP = iron profile (ferritin and transferrin saturation index); **P* ≤ 0.05
