# Supplementary figures and images for: Specific test panels for patients with heart failure: implementation and use in the Spanish National Health System
Source: Adv Lab Med. 2022 Mar 7;3(1):65–70. doi: 10.1515/almed-2022-0006 (PMC10197348; doi:10.1515/almed-2022-0006)

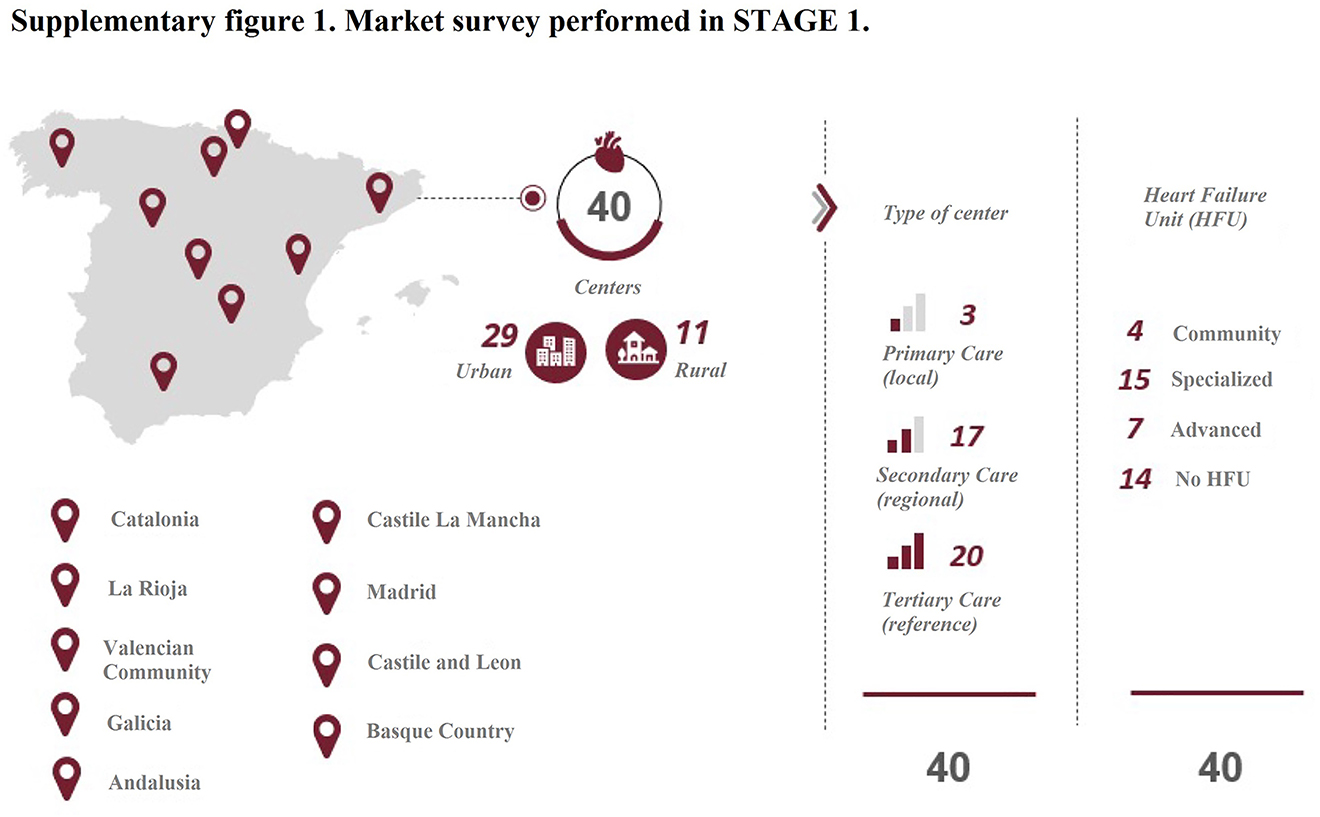

Supplement: Supplementary file 3 — Supplementary Material Details [file j_almed-2022-0006_suppl_003.jpg]

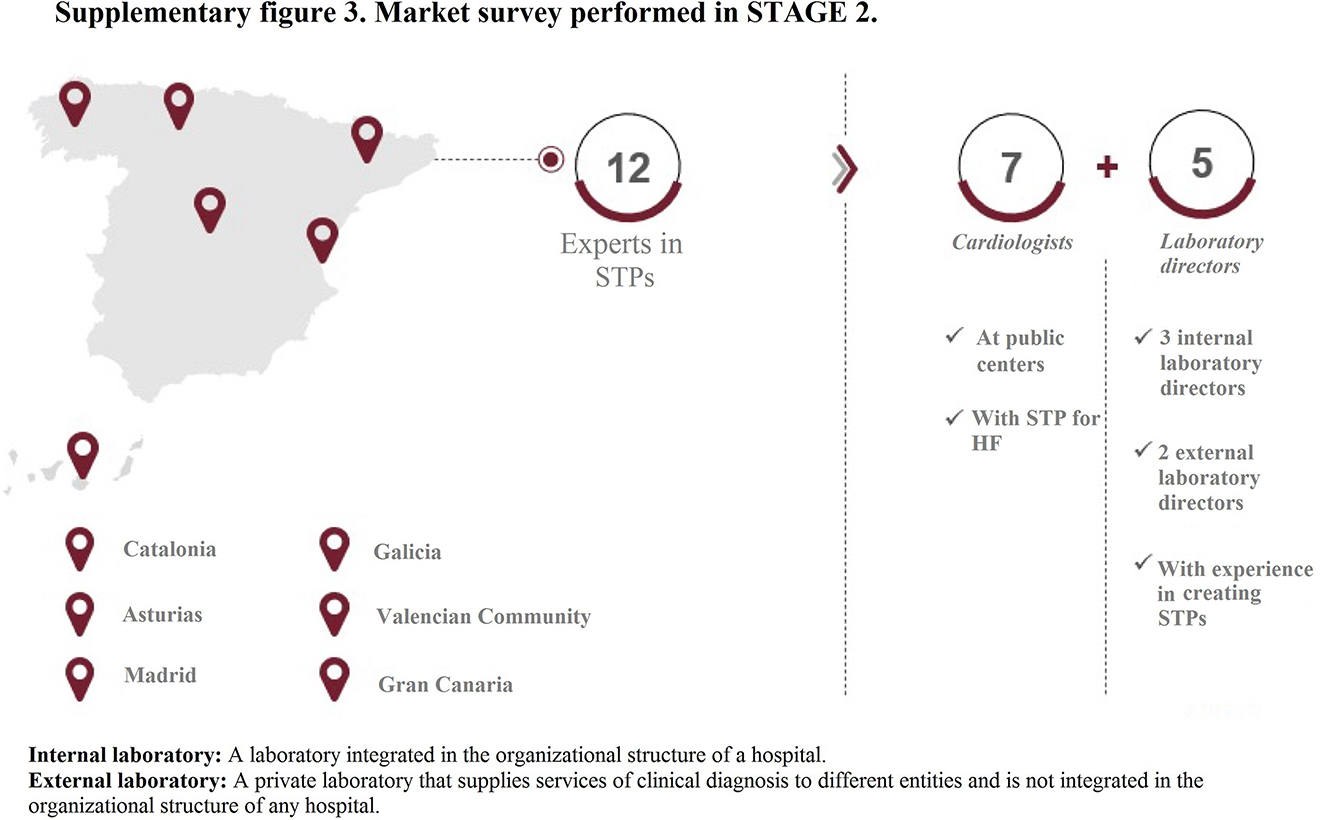

Supplement: Supplementary file 5 — Supplementary Material Details [file j_almed-2022-0006_suppl_005.jpg]
