# Supplementary material for: Specific test panels for patients with heart failure: implementation and use in the Spanish National Health System
Source: Adv Lab Med. 2022 Mar 7;3(1):65–70. doi: 10.1515/almed-2022-0006 (PMC10197348; doi:10.1515/almed-2022-0006)
Supplement: Supplementary file 4 — Supplementary Material Details [file j_almed-2022-0006_suppl_004.docx]

**Supplementary Figure 2. Questionnaire of interviews conducted in STAGE 1.**
0. INTRODUCTION AND PRESENTATION

Good morning,

My name is __________, I work at Anima Consulting. We are conducting a study about the use of specific test panels by cardiologists like you in Spain.

More specifically, we seek your opinion on the establishment and level of implementation of specific test panels in hospitals in Spain. We are evaluating their level of automation, the profile of patients to whom they are applied, etc.

We also want to ask you about aspects related to the measuring of iron metabolism parameters in patients with heart failure (HF).

As you will already know, it is important that you can access your computer during the interview, in case you had to consult your information system for information about the laboratory parameters we are going to talk about.

Do you have any questions before we start?
1. PROFESSIONAL PROFILE

• How many patients with HF do you see per week?

• What percentage of the total of patients do patients with HF represent ?

2. LEVEL OF IMPLEMENTATION OF STP

Great, thank you. Let’s enter into detail on test requests for the diagnosis and monitoring
of HF…

• Are there different types of tests available? What types? Please, mark:
o Standard test

o Extended test

o Other

In case different types are mentioned:

• What is the difference between them?

• What parameters do you request in laboratory analysis orders for patients
with HF?

• Do parameters differ according to patient profile?

• How do you include these parameters in the test request? By selecting them
one by one, using specific test panels…

• In what cases or patient profiles do you use specific test panels for HF?

*Clarification: A specific test panel is a set of parameters that can be selected in a click that*
*include all the parameters the cardiologist wants to measure in a patient with HF.*

o Mark if the participant answers “In all”
▪ Why in all?

o Mark if the participant answers “In some”
▪ In which? Detail:

- XXXX
- XXXX
- XXXX

▪ Why in this type of profiles?
- XXXX

- XXXX

- XXXX

3. TEST REQUEST PROCESS

• How do you order these tests?

• Apart from you, is anyone else involved in the request process?

• Who?

• Are these requests subject to any process of approval?

• What process?

• Are these requests subject to any control system?

• What type of control?

• What information systems do you use to order these tests?

• How long does it take to complete these types of requests? (in minutes)

4. UPDATING OF SPECIFIC TEST PANELS

• Can you edit/update the parameters included in specific test panels?

• How is this process?

• Who is involved in this process?

5. PROCESS OF AUTOMATION

• How long have these processes been automated in your hospital?

• Based on what were these parameters established?

• Could you briefly describe how the automation process was carried out?

• What professionals were involved in this process?

• Do you consider it necessary that iron profile is included in routine tests for

patients with HF?

6. IRON PROFILE (IP)

Perfect, thank you very much, Dr. XXX. Let’s talk about how you diagnose iron deficiency in your office…

• Do you routinely assess the IP of your HF patients?

• What parameters do you usually measure to determine iron profile in patients
with heart disease?

o Ferritin

o Transferrin saturation index (TSI)
o Other

o None

• Are these parameters included in the specific test panels you use?

• If not, how do you include these parameters in test requests?

• From 1 to 10, were 1 indicates “barely” and 10 “very relevant”, how relevant

do you consider that IP is included in routine tests for HF patients?

• What are the advantages and disadvantages of IP being automatically

included?

Thank you very much for your participation, it is very useful for our study.
