# Supplementary material for: Specific test panels for patients with heart failure: implementation and use in the Spanish National Health System
Source: Adv Lab Med. 2022 Mar 7;3(1):65–70. doi: 10.1515/almed-2022-0006 (PMC10197348; doi:10.1515/almed-2022-0006)
Supplement: Supplementary file 6 — Supplementary Material Details [file j_almed-2022-0006_suppl_006.docx]

**Supplementary figure 4. Questionnaire of the interviews performed in STAGE 2.**

**0. INTRODUCTION AND PRESENTATION**

Good afternoon,

My name is __________, I work at Anima Consulting. We are currently
conducting a survey among cardiologists and laboratory directors for a
research study. Our aim is to get an insight into the design process of
test panels in hospitals in Spain.

To collect, record, and analyse this information, we need to take notes
and make audio recordings. Nevertheless, we can stop the recorder
whenever you want. It is important that you feel comfortable during
the interview.

Please, note that this is a confidential study. To guarantee your privacy,
your name and/or any personal information will not be disclosed at any
time.

The purpose of collecting this information is to get a better
understanding of this issue.

We insist that we only seek your opinion and experience. There are no
correct or incorrect answers. Do you have any questions before we
start?

**1. PROFESSIONAL PROFILE AND EXPERIENCE WITH STP**

Firstly, please, let us know you a little better and learn about your
experience with automation processes

• What is your occupation?

• In what centre do you work?

• How long have you worked as a cardiologist in your hospital?
• How do you define “specific test panel”?

At present, there are four request systems for testing iron profile:

1. A system where each iron parameter is selected individually,
without a specific test panel being available (iron parameters are
selected one by one).

2. A system where iron parameters can be selected in block, without a
specific test panel being available.

3. A system of test panels that do not include iron profile tests.

4. A system including test panels, including a test panel for iron profile.

• What type of system is available in your hospital? How long has
it been available?

• Do IM use them too?

• Are these test panels shared with PC? Do GPs use the same

panels?

**2. AUTOMATION PROCESS**

• Were you involved in the automation process? To what extent?

What was your role as a cardiologist?

• If the answer is “no”, have you been involved in other

automation processes? In what processes?

• More specifically, in your opinion, what are the advantages and

disadvantages of this automation? Why?

• In general, how are specific test panels created? Is there any

protocol providing guidelines for the automation of processes?

• If the answer is “yes”, What guidelines does the protocol

provide ?

• If the answer is “no”, How are automation processes arranged?

**3. EXPERIENCE IN THE CREATION PROCESS OF TEST PANELS**

Perfect, thank you very much for this background information. Let’s talk about the creation of test panels for patients with HF/XXXX in which you were involved. Please, describe….

Your experience in the creation of test panels

• How was the experience?

• If you had to describe this experience in a word, what word

would you use? Why?

To get an overview…

• How many phases did the creation of the test panel involve?

• What was your role in each phase? Let the respondent give a

spontaneous response

• What other professionals, apart from you, were involved in each

phase? What was the role of IM? And the role of the laboratory
director?

• What were their duties?

• What were the main motivations for the creation of the test panel?

What were the reasons?

• What were the main difficulties you faced? What were the cause?

• What aspects could have been improved? How?

• What aspects were effective during the creation process? Why?

• How did the presence/absence of a protocol influence the

automation process? Why?

**4. CURRENT USE OF STPs IN PATIENTS WITH HF**

• How long has this test panel been available?

• How often do you use this test panel in the management of HF

patients? (number of times it is used per day).

• From 1 to 10, where 1 indicates “barely” and 10 “dramatically”,

How does this automation process improved the diagnosis of
patients with HF? Why?

• And the treatment of HF in general? Why?

• Why do you think that other centres do not use test panels?

• What are the main barriers to automation?

• How do you think these barriers could be overcome?

• Would you recommend hospitals to create test panels for patients

with HF?

• Why yes/no? (if it depends, what does it depend on?)

• Could you give five reasons why you would do it?

• Who should one talk to encourage the automation of iron profile

testing in other hospitals? Why?

Thank you very much for your participation. It is very useful for us.
